# Book Review

**Published:** 1913-03

**Authors:** 


**Elements of Practical Medicine.** By ALFRED H. CARTER, M.D., M.Sc. Tenth Edition. Pp. xvii, 683. London: H. K. Lewis. 1912. Price 9s. net.—For thirty-two years Dr. Carter's small text-book has held its own. The most striking fact is the way in which the author has kept scrupulously to the very elementary description of diseases which he believes (and many others too) form the surest foundation for a medical student's education. Unlike so many works which have been published in the meantime, Carter's *Medicine* has not made the mistake of trying to inflate itself into a system. We believe that for all its conciseness this little volume contains a very great deal that every student ought to know, and a good deal more than many practitioners remember. In these days of dropsical volumes and a flatulently garrulous letterpress, we grow fonder of the trim khaki-clad veteran of our shelves.

**Acromegaly: A Personal Experience.** By LEONARD MARK, M.D. Pp. viii, 160. London: Baillière, Tindall & Cox. 1912. Price 7s. 6d. net.—An autobiographical account of any disease from the pen of a physician who is himself the subject of that disease forms a contribution to medical literature which must evoke in a particular degree the sympathy of every reader. And this feeling cannot fail to tinge with regret our satisfaction that in this case the description is supremely good. Dr. Mark has avoided the common pitfall of less well-informed writers on their own symptoms, and he has written of acromegaly and its attendant psychical disturbances without that too close self-analysis which betokens true hypochondria. He has given us a restrained but minute account of his sufferings, both psychical and physical, and it cannot be doubted that we are the richer in our understanding of acromegaly by what he has written and suffered. Dr. Mark has brought to the observation of his symptoms not merely the mind of a man well versed in the medical sciences, and full of wider information of a general sort, but in addition that which the study of acromegaly especially demanded, the exceptional faculties of an artist. We are reminded of Gomperz's explanation of the progress made by the Greeks in the lay art of medicine, "Because the physicians of Greece shared with her poets and sculptors the same splendid faculties of keen sight and faithful reproduction of the thing seen." These faculties may be appreciated with lamentable force in Dr. Mark's book, for we fully realise that even as the artistic senses increase, so in like measure must the sensibility to pain and suffering be increased. Putting aside for a moment our personal sympathies for a friend, it is impossible to read these experiences without obtaining a far clearer and more intimate conception of the symptom-complex, which depends upon some unexplained defect of the *sella turcica* and the pituitary body. We may take from Chapter III a marvellous bird's-eye view of the march of events, beginning with slight defects of hearing and sight, and queer feelings in the head, together with drowsiness. Then the miseries of increased sensitiveness to cold and exposure experienced in a country practice. Nasal catarrh, neuralgia and febrile attacks followed, which resembled influenza. Simultaneously indigestion was produced by the altered bite of the teeth. Restlessness seems to have been made harder to bear by reason of an extraordinary sense of fatigue, and presently by attacks of heart failure. Neuralgia, asthenopia, and nasal catarrh all increasing, there came a climax in the disease, and a *black week* with extreme mental depression; and after that a change in the symptoms. The acromegaly began, alterations in physique were noticeable; not, indeed, to the patient, for although his brother discovered the nature of the disease at the age of 45, and Pierre Marie four years later "spotted" him in a crowd at a medical congress as a *typical acromegalic*, it was not until he reached the age of 50 that the knowledge suddenly dawned on Dr. Mark that he was affected with acromegaly. With this knowledge came a decided amelioration of the discomforts of the disease, and the account closes with the remark: "There is still much in life that I am able to enjoy, even if to slight 'unsoundness of eyes' I am gradually adding 'weakness of wind and limb.'" In an afterword Dr. Mark adds one of his characteristic art notes in the form of a "Reverie after contemplating a stone figure of the thirteenth century carved on Reims Cathedral."

**Small-Pox and its Diffusion.** By ALEXANDER COLLIE, M.D. Pp. 58. Bristol: John Wright & Sons Ltd. 1912. Price 2s. net.—It is only natural that the ill-informed householder should view with alarm the establishment of a small-pox hospital in his immediate neighbourhood; but that his fear of infection by distal aërial dissemination is practically, if not absolutely, groundless, is amply shown by Dr. Collie in the careful and painstaking statistics and drawings which he has admirably put together out of his practical experience. It is a proved fact that small-pox is invariably conveyed by the infected person or the infected thing.

**Sprue: its Diagnosis and Treatment.** By CHARLES BEGG, M.B., C.M. Pp. vii, 124. Bristol: John Wright & Sons Ltd. 1912. Price 6s. net.—The principal value of this book is to set forth the author's treatment for sprue by means of santonin. It is widely recognised that this method has a real value, and it is important to follow the author's technique exactly. For instance, recent santonin is almost useless; it must be old and yellow with exposure.

**Vicious Circles in Disease.** By JAMIESON B. HURRY, M.A., M.D. Second Edition. Pp. xiv. 280. London: J. & A. Churchill. Price 6s. net.—Most of the chapters in this monograph have been collated from communications sent to the *Lancet*, the *British Medical Journal*, the *Practitioner*, and the *Medical Press*, and deal with the reciprocal interaction of diseased processes or habits. The contents are arranged under different headings, Nervous System, Cardio-vascular System, and so forth, in good clear type, and the book is in all respects excellently " got up." Many of the vicious circles which the author has carefully described are of great interest, both pathologically and from the point of view of treatment. Some are simplicity itself, such as the fact that scratching inflames eczema and makes it worse; others, in the respiratory and circulatory sections, are more complicated, but all are worked out in a convincing manner, and the value of the information given is very much enhanced by the pertinent and judicious quotations from many well-known English and foreign authors. Most of the circles are medical, but surgery is not forgotten, nor the special senses, and one is struck with the extensive application of the " Zirkelschluss " in every department of our art. The diagrams are good, and illustrate the text in a useful manner. The style is also clear and concise. We may add that in spite of the tabulated nature of this little volume it is very interesting reading. It has, moreover, a good index.

**Manual of Surgery.** By Alexis Thomson and Alexander Miles. Fourth Edition. Vol. II. Pp. xv, 924. Vol. III. Pp. xv, 565. London: Oxford Medical Publications. 1912. Price 10s. 6d. each.—Volume II, dealing with regional surgery, is excellently arranged and beautifully illustrated. New features of the present edition are the exclusion of operative details from the descriptive parts of the work, the former being relegated to a separate volume; the inclusion of a most useful summary of the surgical anatomy of each region at the commencement of every chapter; and the addition of a number of very clear pictures, chiefly taken from actual clinical cases. We have no doubt that it will attain the popularity it deserves. Volume III is a small manual of operative surgery, and represents the appendix to the revised edition of the authors' *Treatise on Systematic Surgery.* It probably has the distinction of being the smallest modern book on the subject, and it will be interesting to note how long its authors will be able to keep it within such narrow limits. It deals with the whole subject from a student's standpoint, giving just enough to enable him to understand the principles concerned, without burdening him with the technical details which he would require to know if he were going to actually perform the operations. Several subjects—such as arterial suture, Matas's operation for aneurism, and the operative treatment of fractures—are described in such a brief way that it may be questioned whether from a practical point of view such description is of any value. The section on cleft palate is one which is so short and incomplete that no student could follow even the general principles of the various methods by reading it. The illustrations are numerous and delightfully clear.

**Die Chirurgischen Untersuchungs-Methoden.** Von Prof. Dr. HUBERT GEBELE. Pp. viii, 192. J. F. Lehmann. München. 1912. (Geheftet M. 8, Geb. M. 9.)—The object of the writer of this book has been to provide a brief, practical, and well-illustrated account of the various diagnostic methods employed in surgery. He does not deal with the clinical symptoms of particular diseases. The earlier sections of the book are concerned with the arts of inspection, palpation and percussion. The next sections are on the modes of passing sounds in various regions and of performing exploratory punctures and incisions; then come sections on the examination of secretions and exudations, and on the diagnostic methods based upon serum reactions. Lastly endoscopy of various kinds and radiography are fully considered. A striking feature of the work is the number and excellence of its illustrations. We may say, in a word, that they have all the good qualities one has learnt to expect in the publications of the firm of Lehmann of Munich. In our opinion the author has supplied a need—the need, namely, for a handy volume containing sufficient information to enable the inexperienced to carry out the various modern methods of examination, instrumental and other, which are essential to the making of an exact surgical diagnosis.

**Leitfaden der Praktischen Kriegschirurgie.** Von Dr. WALTER VON ŒTTINGEN. Pp. xvi, 377. Dresden: Theodor Steinkopff. 1912. Price M. 9.50.—This little book is written as the outcome of the author's practical experience in charge of a red cross field hospital in the recent Russian-Japanese War. It represents a most successful endeavour to summarise the principles of surgery which underlie all the emergencies of battlefield and base-hospital practice. It constitutes a veritable *vade-mecum* for the doctor who volunteers for a war, and who cannot burden himself with a large reference library. The first part deals with general surgery, modern weapons and their missiles, and effects upon their victims; aseptic technique as applicable in emergencies; operations, diet, transport and military sanitation. The second part is devoted to special or regional surgery, detailing the results of gunshot wounds of the various organs, head and extremities, and advising in a short, practical, dogmatic manner as to the best methods of treatment. The book is a wonderful example of its author's power of compressing a vast quantity of practical teaching into a small space.

**The Extra Pharmacopeia.** By MARTINDALE and WESTCOTT. Fifteenth Edition. Two volumes. Pp. xxxi, 1,114; viii, 370. London: H. K. Lewis. 1912. Price 21s. net.—In spite of its thin paper and close printing, the subject-matter of this work has grown to so great an extent that it has been necessary to divide the matter into two volumes. Volume I has been kept down in size to practically that of the last edition. It contains everything the physician and pharmacist are likely to require for immediate reference on therapeutic matters, whilst the second volume acts as a supplement for further study, inasmuch as it contains the analytical memoranda, bacteriological notes, mineral waters, glossaries, analysis of patent medicines, etc. The amount of concentrated useful knowledge on every page is marvellous. In the midst of such wealth it is difficult at times to find what one wants at the moment, but we have no hesitation in saying that the more the book is studied the more useful it is found to be ; in fact, it has proved itself to be indispensable both to the physician who prescribes and to the pharmacist who dispenses.

**Treatment of Neurasthenia by the Teaching of Brain Control.** By Dr. ROGER VITTOZ. Translated by H. B. BROOKE. Pp. viii, 117. London: Longmans, Green & Co. 1911. Price 3s. 6d. net.—This little book is addressed, the author tells us, to those suffering from " neurasthenia, psychoneurosis, or psychasthenia." It is probably very well fitted for its purpose, as it might distract the thoughts of such patients to go through the mental exercises given. To the medical reviewer, however, whom fate compels to read it through, the mental exercise is extremely severe. The central idea is that there are two brains, the " objective " and the " subjective." The latter is the seat of ideas and sensations, the former of judgment, reason and will. The objective normally controls the subjective, but in neurasthenics this control is insufficient or unstable. The diseases mentioned above are essentially a struggle between these two. The treatment is to educate the objective part of the brain. On page 26, however, we come across the statement, " The hand appears to be conscious of anything it touches, but the sensation is obliterated before reaching the brain, as the latter is not sufficiently conscious of what is touched." This checked the little enthusiasm we had to continue, and on pages 30 and 36 the author somewhat suddenly plunges into the " boundless inane," and informs us that the working of the brain can be felt by the physician's hand placed on the patient's forehead. This " working," it seems, gives rise to quite perceptible pulsations or waves, which vary according to the mental effort, and may even be recorded (as on pages 60 and 61) in the form of a diagram. The rest of the book is concerned with the control of these relations by simple mental exercises, and attempts to develop the will power. As hinted above, we can recommend the book to those afflicted with certain neuroses, still more to those who wish to become neurasthenics.

**Landmarks and Surface Markings of the Human Body.** By L. Bathe Rawling, M.B., B.C. Cantab., F.R.C.S. Eng. Fifth Edition. Pp. viii, 96. London: H. K. Lewis. 1912.—The merits of this handbook are attested by the fact that only a year has elapsed since the fourth edition was published. Beyond a few improvements in some of the illustrations the volume remains unaltered. If we may venture a suggestion, it would be to include in the next edition the dates at which the ossification centres of the carpus appear. These most important landmarks in child development are somewhat scantily dealt with in the appendix, although they are readily studied by skiagraphy. Perhaps the author modestly underrates the value of his work for purposes of reference, not only to surgeons but also physicians. In size, type, and illustrations, the book reflects great credit on its publishers.

**Statistics of Puerperal Fever and Allied Infectious Diseases.** By George Geddes, M.D., C.M. Pp. 119. Bristol: John Wright & Sons Ltd. 1912. Price 6s. net.—The object of this book is to show that puerperal fever is due to infection from the hands of those attending the labour, and that this infection comes from previous contamination of the hands in the dressing of suppurating wounds. An immense amount of work has been undertaken in collecting and compiling statistics showing the relative frequency of puerperal fever in the practice of doctors and midwives respectively and the close connection between the puerperal fever rate and the number of accidents occurring in any given area, this connection being the basis of the author's theory that the source of the infection is almost invariably a suppurating wound. The statistics on which the author's conclusions are grounded are most complete, and admirably arranged for easy reference. On this account the book should be of lasting utility to anyone engaged in the study of this subject.

**Clinical Bacteriology and Haematology for Practitioners.** By W. D'Este Emery, M.D., B.Sc. Lond. Fourth Edition. Pp. ix, 274. London: H. K. Lewis. 1912. Price 7s. 6d. net.—Four editions of this practical handbook having appeared since 1902, it is unnecessary to comment at any length on its merits. It was a good book in the first edition, and the author has kept it up to date with his later revisions. The most important addition is a description of the author's simplified technique for the Wassermann reaction. We still know too little about the interpretation of the results of this test to be able to speak dogmatically as to the practical importance of even the original reaction, much less of its many modifications. Dr. Emery makes some useful observations on this subject, based on his experiences during three years. Possibly thirty years hence we may be in a position to speak with greater certainty. Dr. Emery is correspondingly cautious and open-minded.

**Public Health Law.** By W. Robertson, M.D., D.P.H., and A. McKendrick, F.R.C.S. Edin., D.P.H. Pp. xii, 397. Edinburgh: E. & S. Livingstone. 1912. Price 5s. net.—As a book of ready reference and an epitome of law applicable to England, Wales and Scotland, this will prove both handy and reliable. The various enactments are condensed into the smallest possible space, and where necessary the comments on separate clauses are intelligible and instructive, without being too long. Candidates for D.P.H. examinations will find it particularly welcome, especially those seeking for a Scottish diploma.

**Hygiene for Health Visitors.** By C. W. Hutt, M.A., B.C. Cantab., D.P.H. Oxon. Pp. xvi, 415. London: P. S. King & Son. 1912. Price 7s. 6d. net.—As a handbook of ready reference for the now rapidly-increasing class of social workers, qualified and voluntary, this volume will prove most useful. The subject-matter is well arranged, clearly illustrated, and embraces most topics of importance. The section dealing with the treatment of ophthalmia of the newly born might well have been placed in the chapter on the care of infants and young children. The sterilisation, rather than the boiling of milk, we think should be recommended for the infant, and we are doubtful as to the wisdom of giving a child who is being successfully breast-fed a midday bottle feed of cow's milk and water, merely to facilitate its weaning later on. A paragraph on the house fly and the danger arising from the spread of infection through its agency might appropriately be added to the chapter on the prevention of diseases. For those anxious to qualify as successful health visitors the book will be an indispensable *vade-mecum.*

**On Gastroscopy.** By William Hill, B.Sc., M.D. Lond. Pp. vii, 46. London: John Bale, Sons & Danielsson, Ltd. 1912. Price, 3s. 6d. net.—This short monograph is an expansion of a paper read at the Birmingham Annual Meeting of the British Medical Association in 1911. It contains an account, copiously illustrated, of the evolution of the various present-day methods of gastroscopy, and particularly a description of the instruments elaborated by the author, in conjunction with Dr. George Herschell, for œsophago-gastroscopy by combined direct and indirect (periscopic) vision. Also the methods of using the latter instruments, the dangers to be avoided, and some of the successful observations made with them, are detailed. We cordially recommend a perusal of the work to all practitioners, for the author makes it clear that valuable data for the diagnosis and treatment of gastric disorders are obtainable with a minimum of risk by the gastroscopic methods he describes.

**Stomatology.** By H. P. Pickerill. London: Oxford Medical Publications. 1912. Price, 15s. —This book is intended to cover the field where general surgery and dentistry meet, and in that respect it meets a real need. It describes for the benefit of the medical man the deformities of the jaws, the causation, prevention and treatment of dental caries, the treatment of fractured jaw, and the dangers of oral sepsis. For those interested in the subject no better book can be recommended.

**The Essentials of Food.** By Donald Stewart, M.D. London: John Bale, Sons & Danielsson, Ltd. 1911. Pp. vi, 182. Price, 3s. 6d. net.—The author of this small book attempts to give a "correct summary of present-day knowledge about foods" in language which can be understood by those who have no special scientific or medical training. The subject is a large one, and modern physiological chemistry is a difficult thing to put into untechnical language. Few men, indeed, except Huxley, have ever succeeded in the task of divesting a specialised science or scientific problem of its technicalities so that the uninformed can profit thereby. Dr. Stewart's attempt we cannot label a success. It is too evident that he has not sufficient knowledge of the subject at first hand to render his book of much value. He quotes many tables, and takes considerable pains to enforce certain precepts tending towards vegetarianism, but the language is often confusing and the statements inaccurate. He says, for example, that "concentrated preparations of animals" have "no value as nutritives," and refers the reader to an "analysis of these" (p. 31). On referring to p. 31 we find an analysis of eggs. He talks about a "gorged liver" and a "loaded tongue," about a "*preter-natural* accumulation of waste products," and on p. 13 he gives us the formula for "hair," which the reader will be glad to know is CHNOSuSi. The composition of sugar is CHO, and we are instructed that in the formula $H_2O$ "the figure 2 under the H means not two 'atoms' of the hydrogen, but two elements have to be taken with one of oxygen to form water." The physiologist is spoken of more with pity than anger. Of the digestion of milk he remarks, "There may be thus two bodies present in the stomach at the same time, and it is this combination that creates the misunderstanding in the minds of physiologists." What the state of mind of a physiologist might be after an ordinary dinner we tremble to think! The specific gravity of cow's milk is given as "10.32 per cent.; whey—same." Such statements as this, "Proteid means that the substance is of the highest value as a food, perhaps albumen comes next," show the nature of the book.

The reviewer, however, might forgive all this as a pleasant comic interlude ; but when on p. 128 Dr. Stewart, speaking of alcohol, says, " In health it is so dangerous that like the devil in *Paradise Lost*, 'farthest from him is best,' " he feels that an apology is due to the shade of Milton for so bad a misquotation about one we had best treat with respect.

**The Care of the Skin in Health.** By W. ALLAN JAMIESON, M.D., F.R.C.P. Eng. Pp. 109. London: Henry Frowde, Hodder & Stoughton. 1912.—It is evident that this book is intended for readers outside the medical profession, and is couched in very general terms. So far as it goes it is excellent, there is much good advice on washing of the skin, bathing, and care of the hair and nails. In fact, it seems to represent a tentative excursion into fields which hitherto have been considered unworthy the serious attention of dermatologists, and the lawful province of beauty specialists, cosmetic quacks, and institutes of physical culture. We should like to see Dr. Jamieson bring his experience and knowledge rather more boldly into the lists, and claim " cosmetics " as a genuine branch of dermatology. The best way to abolish quackery is to excel the quacks in wisdom and understanding. The type from which this book is printed is exceptionally good, and makes very pleasant reading.

**Extraction of Teeth.** By J. H. GIBBS, F.R.C.S., L.D.S. Edin. Pp. 163. Edinburgh: E. & S. Livingstone. 1912. 7s. 6d. net. —This is an interesting book, expressing the views of the author, who considers that many of the orthodox methods of extracting teeth are wrong in principle and bad in practice. Mr. Gibbs describes his operative technique clearly and fully, and while there will be diverse opinions on many of the points raised, we are in accord with the author in his plea for the use of as few and simple forms of forceps as possible. There is a short but practical article on disinfection, and also on the choice of an anæsthetic for dental operations. The chapter on difficult extractions, which includes notes of many cases, and some good illustrations, is excellent. We commend this book to all who are interested in the subject.

**An Historical Cutline of Ambulance from the Earliest Times.** By C. H. MILES, L.R.C.P. (Lond.). Pp. 24. Bristol: John Wright & Sons Ltd. 1912. Price 3d.—Dr. Miles has published this brief historical account of ambulance work in the belief that it will add to the interest taken in this subject by members of " First Aid " and similar organisations. A few notable illustrations of military surgery in Homeric times combined with a note on the methods of the Roman army suffice for antiquity; medieval times contribute two or three more examples of "the science of ambulance surgery" and lead up to a short description of the origin of the "Hospitallers of St. John of Jerusalem" and the ultimate development of the Order of the Hospital of St. John of Jerusalem in England. The only fault we have to find is that the history of so interesting a topic should be so aggravatingly short.

**St. Bartholomew's Hospital Reports.** Vol. XLVII. London: Smith, Elder, & Co. 1912. Subscription price 6s.—This year's reports have a sad note in that they record the death of three well-known late members of the staff, Langton, Power, and Gee. The appreciations by D'Arcy Power, W. H. Jessop, and Norman Moore are well written and give excellent portraits. Amongst the medical papers one by J. A. Nixon is especially worth reading, being a careful epitome of many cases of abdominal aneurysm. There is also an interesting thesis on "the causes of disease at sea in the Tudor and Stuart periods," by Alan Moore. McAdam Eccles contributes a fourth series of cases of Intussusception.

**The Bristol Eye Dispensary, 1812–1912 (A Retrospect).** By Edgar A. Prichard. Pp. 20. Bristol: J. W. Arrowsmith Ltd. Price 3d.—Last year was the centenary of this institution, and to commemorate the event Mr. Prichard has written a small illustrated pamphlet giving an interesting history of the Charity since its foundation. The chief feature of the place is the Sunday morning clinic, which was established in order to avoid many poor patients losing a day's work; and the dispensary has been open every single Sunday since it was opened in September, 1812, and it has gone on quietly doing good work and receiving great numbers of patients now for a hundred years, and yet there are many who have scarcely even heard of it. It is probably the most economically run institution in the kingdom, and we think it well worthy of support.

**An Operating Theatre in Private Practice.** By C. Hamilton Whiteford. Pp. 76. London: Harrison & Sons. 1912. Price 3s. 6d. net.—To those contemplating the building of a private operating theatre this book offers many useful hints. One doubts the advantages of the south aspect advocated, knowing how intolerable even a north light may become in hot weather, but it should certainly minimise the cost of heating, which in the theatre described is by electricity, and deserves special consideration, for if really efficient it is a marked improvement, from an aseptic point of view, on the radiators in common use. The author in his own case seems to have succeeded in producing a theatre which provides all the requirements demanded by modern aseptic technique.

**Deuxième Congrès de l'Association Intérnationale d'Urologie.** Londres. 24–28. Juillet, 1911. London: Adlard & Son. 1912. Price 10s. net.—In these proceedings will be found a most valuable collection of reports and discussions on the present position of the treatment of bladder growths. The ultimate results of prostatectomy are fully discussed, and it contains probably the best series of contributions on phosphaturia in the literature.

**Manual for Women's Voluntary Aid Detachments.** By P. C. Gabbett, Lt.-Col., I.M.S. Pp. 103. Bristol: John Wright & Sons Ltd. (N.D.). Price 1s. net.—This is a useful book, and a marked contrast to some already published on "First Aid." A careful study of it will make women's detachments a great boon to the sick and wounded in the time of war.

**Guy's Hospital Reports.** Edited by F. J. Steward, M.S., and Herbert French, M.D. Vol. LXV. London: J. & A. Churchill. 1912.—The volume opens with a statistical paper on "The Outlook of Sufferers from Exophthalmic Goitre," by Dr. W. Hale White, giving the results of 124 cases of exophthalmic goitre met with in hospital and private practice, so as to try to form some opinion as to the outlook for sufferers from this disease. The result of the observations goes to indicate that the expected mortality of patients not operated upon is about twice as great as it should be according to the "Healthy Females Experience of Twenty British Offices amongst Assured Lives." Another paper on "The Operative Treatment of Exophthalmic Goitre," by L. Bromley, M.B., B.C., gives particulars of six cases of operation in the wards of Guy's Hospital, with two deaths and four recoveries. Mr. Bromley also gives an able summary of the advantages and risks of operation for this disease, pointing out that "operative treatment, in carefully selected cases, by an experienced operator is a justifiable proceeding," which seems rather to damn by faint praise. Perhaps the most interesting paper in the present volume is one by Arthur F. Hertz, M.A., M.D., etc., entitled "Neurological Studies," under which heading he writes on such subjects as The Tendo-Achilles Jerk, Babinski's Second Sign, Unilateral Musculo-Spiral Paralysis or Saturday Night Paralysis, etc. There is now at Guy's a Neurological Department, which must present a large field for observation, and evidently Dr. Hertz and others associated with him have taken full advantage of the opportunities afforded them to study these fascinating subjects. A prize essay of the Pupils' Physical Society, by Dr. Alexander Sandison, on "Rheumatic Fever in the Last Decade," gives a very complete *résumé* of recent advances in the study of acute rheumatism, and is accompanied with an analysis of the cases admitted to Guy's Hospital during the period from 1900 to 1909, during which period we see the rise, growth, and to a certain extent the fall of the hypothesis of a specific micro-organism as the specific causal agent. The author truly says that among expert bacteriologists there is so much contradictory opinion that the inexpert, before pledging himself to any particular hypothesis, must be content to await future developments. Other notable papers in the present volume include one by Dr. A. S. Morton Palmer on "Observations on the Deep and Surface Temperatures of Man in Health and Disease," and one on "The Administration of Radium and its Derivatives with Reference to their Possible Application to Cancer," by Dr. E. Bellingham Smith and Mr. W. Wilson. Various lists of school appointments, prizes, etc., make up a volume of great interest to all old Guy's men.

**A Surgical Treatment of Locomotor Ataxia.** By L. N. Denslow, M.D. Pp. ix, 118. London: Baillière, Tindall and Cox. 1912.—Locomotor ataxia is considered to be due in most cases to a peripheral nerve irritation in the urethra in a subject whose nervous system has been rendered susceptible to injury by syphilis. Not only can strictures and granulating surfaces be discovered in the urethra of such patients, but on treatment of these conditions by dilatation and local applications the spinal symptoms are rapidly ameliorated. The number and variety of diseases which are mentioned as arising directly or indirectly from urethral irritation reminds one of that system of fanciful gynæcology which attributes most of the ills that female flesh is heir to to uterine displacements. Probably the one system of pathology and treatment deserves as little serious attention as the other.

**Common Disorders and Diseases of Childhood.** By G. F. Still, M.A., M.D. Cantab. Second Edition. Pp. xiv, 813. London: Oxford Medical Publications. 1912.—The present reviewer in surveying the first edition of Dr. Still's book felt constrained to recommend it to every practitioner of medicine. It is an admirable superstructure of common sense, built up on the solid foundation of personal experience; a work of which British medicine may well be proud. Dr. Still has added several new chapters, and made important changes in some of the others. We note with interest that he thinks urotropin worthy of trial in the early stages of acute poliomyelitis, and that he regards the use of salvarsan in congenital syphilis as unnecessary and therefore unjustifiable. Of the new chapters, that dealing with tonsils and adenoids may be specially mentioned, although all of them are most interesting and instructive.

**Cerebral Decompression in Ordinary Practice.** By C. A. BALLANCE, M.V.O. Pp. 71. London: Macmillan and Co. Ltd. 1912.—The substance of this brochure was delivered at a meeting of the Bath and Bristol Branch of the British Medical Association in February, 1912. The author deals with the subject under two headings (*a*) Decompression for Injury; (*b*) Decompression for Disease. The value of decompression operations in preventing impending death from cerebral compression, or in preventing blindness from similar causes, is established, and though as the author shows one must not expect too much, on the other hand he makes it clear that in suitable cases the method is of undoubted utility.

**Materia Medica and Therapeutics.** By J. MITCHELL BRUCE, M.D., and WALTER J. DILLING, M.B. Ninth Edition. London: Cassell & Co. Ltd. 1912.—The first edition of "Mitchell Bruce" was published in 1884, and that and subsequent issues have served as a guide to vast numbers of medical students through the devious pathways of materia medica and allied subjects. The present and ninth edition is introduced jointly with Mr. Walter J. Dilling, M.B., Lecturer in Pharmacology at Aberdeen University. The subject-matter has been carefully revised, and notes on the more recently introduced synthetic remedies have been added. At the present time the study of physical chemistry has reached such a point that it cannot be ignored by students of medicine, and it is refreshing to notice that ionic medication is discussed—rather too briefly—but this is sufficient to indicate that materia medica is gradually being reformed and brought into line with modern thought. This progress is also evident when several pages of this work are seen to be devoted to notes on "Vaccine-Therapy."

There is room for improvement in one direction: it would be more convenient if the structures of many of the synthetic compounds were expressed "graphically," as the student can form far better mental pictures of the constitution of drugs when the formulæ are so represented. As a concise handbook for students and others we can safely recommend the work.

**What to do in Cases of Poisoning.** By WILLIAM MURRELL, M.D., F.R.C.P. Eleventh Edition. Pp. 283. London: H. K. Lewis. 1912.—The need for eleven editions in twenty-two years is sufficient to indicate the value of this little work. The last has been thoroughly revised, and many additions have been made, including the deadly veronal, the use of which has been enormously increased with frequent fatal results. It appears to have been overlooked and not included in the Schedule of Poisons Act, 1908, so that it is easily obtained without a prescription, is tasteless, and fairly soluble.

**The Cause of Cancer, being Part III of "Protozoa and Disease."** By J. JACKSON CLARKE, M.B., F.R.C.S. Pp. xi, 112. London: Baillière, Tindall & Cox. 1912.—Mr. Jackson Clarke has put into book form his views upon the relation of protozoa to disease. He recounts his experiences at the hand of the Morbid Growth Committee of the Pathological Society of London, and pleads for a fuller consideration of the hypothesis he advances. Although the protozoa are not deemed by many to play an important part in the ætiology of cancer, their capacity to act as irritants is universally recognised, and the author has the satisfaction that he has worked in a field which has yielded many surprises, and furnished the basis for much of our present-day therapy. It must be left to the future to determine whether the cancer cells or their inclusions are parasitic as the word is usually understood, but we cannot fail to recognise the persistence and perseverance which Mr. Clarke has shown in his work. To such persistence and enthusiasm Nature often yields her secrets.

**The Pollution of Swimming Baths.** By J. GRAHAM FORBES, M.D. Pp. 15. London: J. & A. Churchill. 1912.—The results of a bacteriological investigation of the water of one of the London County Council School Swimming Baths impressed the author with the evils and dangers lurking in swimming baths as a means of conveying infection. This brief paper discusses a subject of great importance, and the references to similar investigations by other observers add to its value. The author lends his support to Dr. Alexander's advocacy of sterilisation of swimming baths by the use of an electrolytic disinfecting fluid.

**Wellcome Photographic Exposure Record and Diary. 1912.** Pp. 280.—On comparing the copy for this year with that of 1911, we find that the changes are few, but the *Record* is so complete in every detail that the photographer can require that yearly changes are next to impossible. The set of standard pictures is, we think, a better one this year than in previous ones, and the list of plates and films has been brought up to date by the latest additions of the makers. This is the ninth year we have used the *Wellcome Record*, and we hope never to be without it.

**The Illness and Death of Napoleon Bonaparte.** By ARNOLD CHAPLIN, M.D. Cantab., F.R.C.P. Pp. 112. London: Hirschfield Brothers Limited. 1913. Price 2s. 6d.—This medical criticism of the illness and death adds one more essay of over a hundred pages to the voluminous literature of the life and death of the exile of St. Helena. Political considerations were responsible for much of the misconception which arose as to the nature of the malady. The British Government on the one hand and the Emperor's *entourage* on the other were at such variance that the truth (lying somewhere near the mean) has been difficult to fathom. The facts now justify with tolerable certainty the opinion that the cancer which killed Napoleon was secondary to a chronic ulcer, from which he must have suffered for a considerable time, and in the edges of which it originated. From the medical standpoint it is easy to realise what the difficulties of diagnosis between chronic ulcer, simple or malignant, hepatitis, hypochondriasis, and climatic influences must have been. The oft-repeated statement that Napoleon Bonaparte must be classed as an epileptic appears to have absolutely no foundation.

**Manual of Medicine.** By A. S. Woodwark, M.D., M.R.C.P. Pp. xi, 409. London: Oxford Medical Publications. 1912. 10s. 6d. net.—This is a "cram" book pure and simple, and one of the best of its kind. An enormous amount of information has been crowded into a very small space. The book should prove extremely useful to those revising their work previous to examinations, and will also appeal to the student just entering the medical wards, who is anxious to read up his cases. Leading features are the alphabetical arrangement of the infective fevers, and the indication in the table of contents of the relative importance of the subjects treated by the size of the type employed: in the latter connection it is somewhat astonishing to find appendicitis printed in the smallest tpye. The various causes of many leading *symptoms* are enumerated in lists. As a synopsis of the subject, the section on diseases of the nervous system is particularly good. Where so much compression has been attempted it is, perhaps, inevitable that some parts should strike a reader as unduly "thin." We think that in future editions the paragraph dealing with appendicitis, especially under the heading of "Operation," and that on tuberculous laryngitis might well be somewhat expanded.

**Clinical Medicine.** By Judson S. Bury, M.D., F.R.C.P. Edited by Judson S. Bury and Albert Ramsbottom, M.D. Third Edition. Pp. xxi, 530. Ten plates. London: Chas. Griffin & Co. 1912. 17s. 6d. net.—The third edition of this well-known book has been revised, and many additions and alterations made to render it more useful. It may be recommended as a trustworthy guide and a useful aid to clinical work. There is an excellent and well-illustrated section on the examination of the thoracic and abdominal organs by the Röntgen Rays, a subject of rapidly-growing importance. We can also commend the section on skin diseases. The book is very complete, clear and well written, and the illustrations numerous and helpful. Any criticisms we have to offer are of a minor character: thus, in taking a culture from the blood there is no need to use the piston of a glass syringe, as if the needle is in the vein the blood will flow into it, also a little 0.5% citrate of soda solution in the syringe prevents too rapid coagulation. The plates representing blood films are good, except that the colours of the nuclei do not exactly represent that produced by Leishman's stain. We think that the lying instead of the sitting posture of the patient should be advised for lumbar puncture, because so many patients in whom it is required cannot sit up. The temperature variations in apoplectic seizures should be given. On looking up the reference to Wassermann's test in the index we could not find it on the page indicated, nor could we find any description of this test. Lastly, we could not find any account of the newer tests in the diagnosis of vertigo. We mention these points in the interests of the future editions which we hope will reward the author's well-directed labours.

**Pye's Surgical Handicraft.** Edited and largely rewritten by W. H. Clayton-Greene, B.A., M.B., B.C., F.R.C.S. Sixth Edition. Bristol: John Wright & Sons Ltd. 1912. 12s. 6d. net.—In its present form this work is one of the most practical books on minor surgery. It has been largely rewritten, and considerable care appears to have been exercised in bringing it up to date. It can be thoroughly recommended for general practitioners, dressers, and the immature house surgeon.

